# Interweaving Disciplines to Advance Chemistry: Applying Polyoxometalates in Biology

**DOI:** 10.1021/acs.inorgchem.1c00125

**Published:** 2021-03-31

**Authors:** Nadiia I. Gumerova, Annette Rompel

**Affiliations:** Universität Wien, Fakultät für Chemie, Institut für Biophysikalische Chemie, Althanstraße 14, Wien 1090, Austria

## Abstract

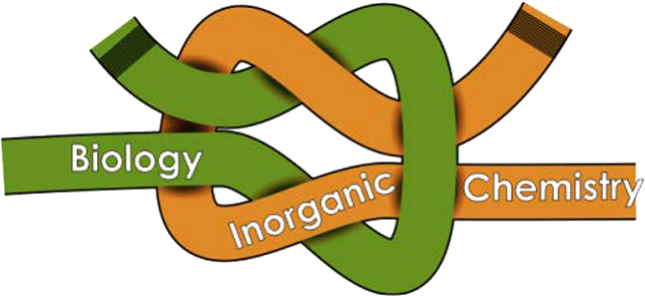

This Viewpoint brings awareness of the challenges and subsequent breakthroughs at the intersection of different disciplines, illustrated by the example of the influence biological entities exerted on a huge class of inorganic coordination compounds, called polyoxometalates (POMs). We highlight the possible effects of biological systems on POMs that need to be considered, thereby emphasizing the depth and complexity of interdisciplinary work. We map POMs’ structural, electrochemical, and stability properties in the presence of biomolecules and stress the potential challenges related to inorganic coordination chemistry carried out in biological systems. This Viewpoint shows that new chemistry is available at the intersections between disciplines and aims to guide the community toward a discussion about current as well as future trends in truly interdisciplinary work.

## Abstract 2

We discuss the investigation of polyoxometalates in biological systems as one future direction of chemistry. Highly interesting, new, and sometimes spectacular findings and applications can be obtained from correctly carried out interdisciplinary research. In this Viewpoint, the challenges of truly interdisciplinary work and concepts for overcoming boundaries while working on intertwining disciplines are discussed.

## 1 Introduction

The division of our knowledge into faculties is done by scientists who are seeking new knowledge often in increasingly highly qualified and specialized scientific disciplines. Nature, in contrast, knows no faculties or work that is limited to artificially defined areas of competence. Working within the boundaries of traditional scientific disciplines is too rigid to adequately address many of the current real-world problems we face, including economic development, climate change, and overcoming systemic diseases. Scientists are therefore encouraged to undertake multidisciplinary, interdisciplinary, and even transdisciplinary work in order to solve today’s most challenging and complex problems in ambitious partnerships and by bundling multidisciplinary knowledge and expertise.^1,2^ Multidisciplinarity describes the phenomenon of people from different disciplines working together, each drawing on their own disciplinary knowledge, interdisciplinarity describes the integration of knowledge and methods from different disciplines using a real synthesis of approaches, and transdisciplinarity describes a phenomenon that creates a new unity of intellectual structures beyond the disciplinary perspective.^3^

Studies at the interface of chemistry and biology are a prominent example of going beyond established areas of competence and thereby producing exciting new research results of the utmost importance. In the last few decades, we have made important advances in our knowledge of mechanistic biological chemistry and steadily broadened the traditional view of the different roles that metals and their complexes play in biological systems because they are increasingly discussed at various conferences (Metallomics, Metals in Biology, International Conference on Biological Inorganic Chemistry, etc.). The study of the interactions between inorganic complexes and biomolecules is a prime target of multidisciplinary research. In this perspective, we show unpredictable and unprecedented discoveries that result from the study of polyoxometalates (POMs) in biological systems and are the result of highly interdisciplinary work. The examples show that some off-type chemistry could so far only be observed at the intersection with other disciplines, whereby the POMs–biomolecules investigation is only one example that can be extended and transferred to other branches of research.

Since 1970, when the inhibitory effect of the silicotungstic acid H_4_[Si^IV^W^VI^_12_O_40_] on murine leukemia and sarcoma viruses was discovered,^4^ the application of POMs in various biological systems has been a rapidly growing branch of science. The number of papers on POMs published in the context of biological applications has tripled in the past decade (from 115 publications in 2009 to 443 in 2019, based on a search in the Scopus database in August 2020 for the term “polyoxometalate AND application” and then limited on the scientific disciplines “Biochemistry, Genetics and Molecular Biology”, “Pharmacology, Toxicology and Pharmacy”, “Medicine”, “Agricultural and Life Sciences”, and “Immunology and Microbiology”). A large number of studies have been conducted on the antiviral^5^ and insulin mimetic effects,^6^ as well as on the anticancer^7^ and antibiotic^8^ activities of various POMs. POMs inhibit a number of biological processes, such as β-amyloid aggregation^9^ or the activity of P-type ATPase,^10^ and can even act as artificial enzymes.^11^ In order to contribute to the understanding of the role POMs play in biological systems, a multi- and interdisciplinary approach is required because POMs, like other inorganic coordination compounds, are flexible and reactive molecules, and their identity and integrity are highly dependent on the reaction conditions, such as the pH value, the type of buffer used, and influences from other components in the medium,^12^ to name just a few. The inclusion and evaluation of the biological activity of POMs requires in-depth inorganic, biochemical, and biological knowledge.

In this Viewpoint, we use the application of POMs in biological systems as one example to show that highly interesting, new, and sometimes spectacular findings and applications can be obtained from correctly carried out multidisciplinary, interdisciplinary, and transdisciplinary work. However, this assumes that researchers contribute more than just their own expertise and are ready for an interdisciplinary discourse, are aware of the challenges connected with interdisciplinary work, and value the gained knowledge highly enough. The following examples are collected from the literature, to which the authors of this Viewpoint also contributed.

## 2 Findings at the Interface between Disciplines

### POM Species in Physiological Buffers and Culture Media and in the Presence of Biomolecules

Biological buffers are sometimes viewed as minor actors, and their important role is often underestimated. Because the behavior of metal oxides is highly dependent on the pH value and the POM’s hydrolytic stability is limited to a certain pH range depending on its archetype, the buffer does influence the stability range.^12^ The presence of macromolecules can significantly affect the POM’s stability, as demonstrated on the decavanadate [V^V^_10_O_28_]^6–^, the best studied POM anion under biological conditions.^13,14^ At pH 7.5, the decavanadate’s half-life can be increased 5.5-fold in the presence of G-actin,^14,15^ while the addition of *Mycobacterium smegmatis* or *Mycobacterium tuberculosis* cells to a [V^V^_10_O_28_]^6–^ solution between pH 5.8 and 6.8^13^ causes immediate decomposition of the decavanadate. Note that [V^V^_10_O_28_]^6–^ by itself does not show hydrolysis in solution between pH 5.8 and 6.8.^16^ Changes in the hydrolytic stability caused by the addition of G-actin^14^ or *M. smegmatis* and *M. tuberculosis* cells^13^ contradict the well-accepted stability behavior of POMs in pure inorganic media, and the observed changes in POM’s properties through the addition of biomolecules are often unexpected for inorganic chemists. The POM’s behavior observed in the presence of biomolecules can, of course, be applied to other applications in solution. For example, the addition of a biomolecule to a POM solution could be considered to be advantageous if a specific POM anion needs to be kept stable over a certain pH range where it would not be stable under purely inorganic conditions.

### Reduction of POMs Caused by Biomolecules

**Figure 1:**
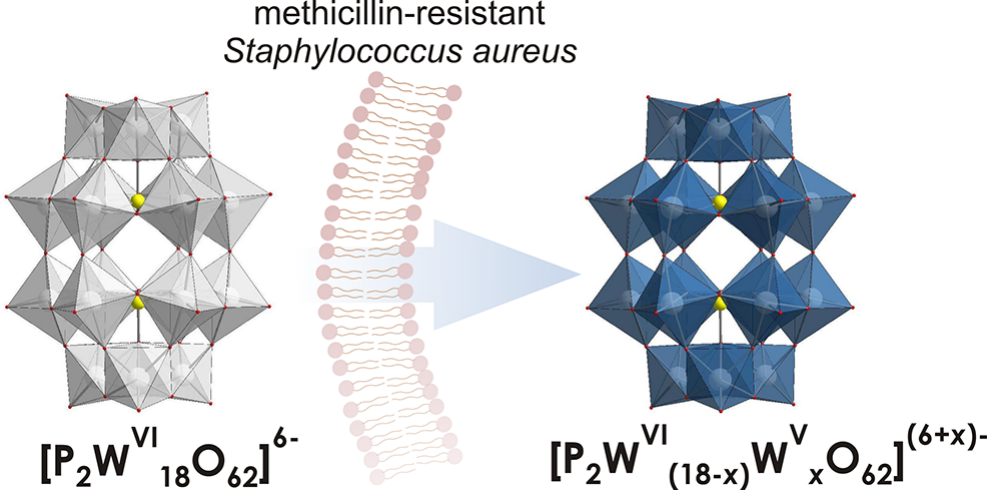
Penetration of the Wells–Dawson POT [P^V^_2_W^VI^_18_O_62_]^6–^ through the MRSA cell wall accompanied by reduction to [P^V^_2_W^VI^_18–*x*_W^V^_*x*_O_62_]^(6+*x*)–^, tracked by a color change from colorless to blue.^19^ Color code: {WO_6_}, gray in oxidized form and blue in reduced form; O, red; P, yellow.

While synthetic chemists like to use very strong inorganic reducing agents such as B_2_H_6_, NaBH_4_, N_2_H_4_, and NH_2_OH to synthesize electron-rich anions,^17^ reduction of POMs in the presence of reducing biomolecules is already observed under “milder” conditions and often POMs remain reduced for at least hours.^18^ Reduced polyoxomolybdates (POMos) and polyoxotungstates (POTs) are very easy to recognize because of their blue color, which results from the intervalence charge transfer of M^V^–O–M^VI^ ↔ M^VI^–O–M^V^.^17^ The uptake of the Wells–Dawson POT K_6_[α-P^V^_2_W^VI^_18_O_62_] (**P**_**2**_**W**_**18**_) into methicillin-resistant *Staphylococcus aureus* (MRSA) cells could be impressively observed through the color change from yellow to blue due to POT reduction (Figure 1).^19^ The protoplasts of the blue-stained MRSA cells remained blue for at least 12 h, which suggested the penetration of a reduced POT [α-P^V^_2_W^VI^_18–*x*_W^V^_*x*_O_62_]^(6+*x*)–^ through the cell wall. For the POT reduction in MRSA cells, the authors postulate that **P**_**2**_**W**_**18**_ enters the respiratory electron-transfer system NADH/ubiquinone/cytochrome *c*, whose components have a sufficiently negative redox potential (NADH, −0.96 V vs Ag/AgCl; ubiquinone, −0.54 V vs Ag/AgCl; cytochrome *c*, −0.39 V vs Ag/AgCl) to reduce **P**_**2**_**W**_**18**_ (−0.05 to −0.85 V vs Ag/AgCl^20^).

The novelty and unpredictability of the POM chemistry in this case does not lie in the fact that reduction has taken place in the complex, reducing-agent-containing cells in the media, but in the period during which the POT remains electron-rich in the biological environment. While a constant supply of reducing agent in a living cell supports a reduced state of POMs, in inorganic solutions, POTs are, unlike POMos, easily reoxidized by atmospheric oxygen.^17^ Because of the ability of POMs to undergo a large number of redox processes in a reversible fashion, they have been investigated as potential energy storage devices.^21^

### Exciting POM Structures in the Presence of Proteins

The properties of POMs can be changed completely by adding just one {MO_6_} (M = addenda ion) octahedron, which is shown by a comparison between the Lindqvist POT [W^VI^_6_O_19_]^2–^ and the heptatungstate [W^VI^_7_O_24_]^6–^. The Lindqvist-type [W^VI^_6_O_19_]^2–^ is only stable and redox-active in organic solvents, while the heptatungstate [W^VI^_7_O_24_]^6–^ is stable and redox-inactive in aqueous solution.^22^ The synthesis of new POM archetypes is the driving force behind the POM chemistry, whereby a small change often opens up new avenues for POM development.

**Figure 2:**
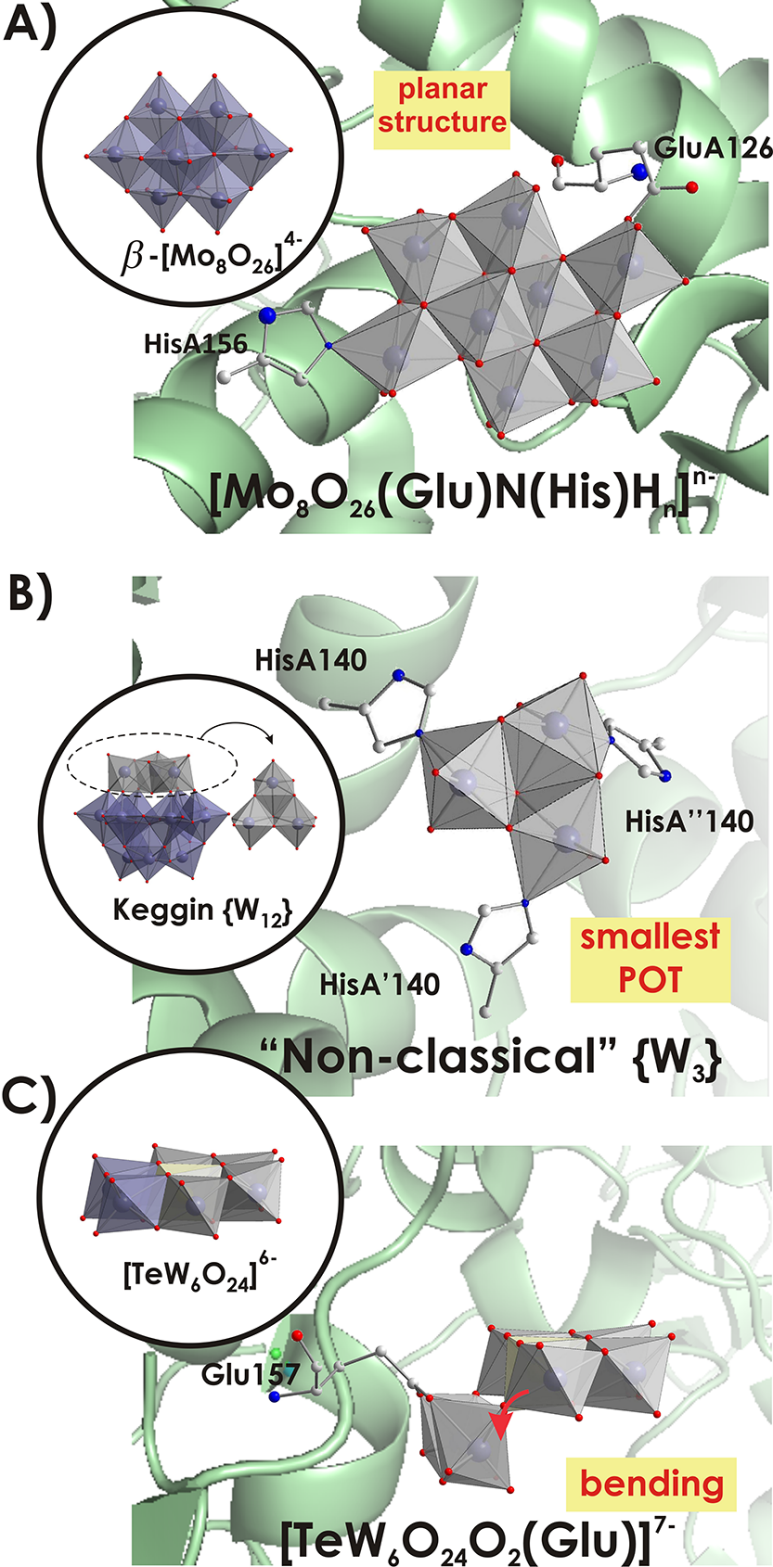
“Nonclassical” and classical POMos and POTs. (A) “Nonclassical” {Mo_8_} was found in the MoSto protein from *A. vinelandii*, and β-[Mo^VI^_8_O_26_]^4–^ could be crystallized from a Na_2_Mo^VI^O_4_ solution acidified to a pH lower than 5.^23c^ HisA156 and GluA126 are histidine and glutamate side chains. Color code: {MoO_6_}, gray or purple; C, gray; N, blue; O, red. (B) “Nonclassical” [W^VI^_3_O_10_H_*x*_N_3_]^(6–*x*)–^ {W_3_} was found in the WSto protein from *A. vinelandii*; metatungstate [H_2_W^VI^_12_O_40_]^6–^ {W_12_} with its {W_3_} could be synthesized from a solution of Na_2_W^VI^O_4_ acidified to pH ∼ 5.^23d^ HisA140, HisA′140, and HisA″140 are histidine side chains from three α subunits. Color code: {WO_6_}, gray or purple; O, red. (C) Covalent binding of TEW^24^ to the polyphenol oxidase *cg*AUS1.^25a^ The carboxylic O atoms of glutamic acid (Glu157) bind covalently to two W atoms of TEW, accompanied by a rearrangement within the Anderson–Evans structure resulting in a bent structure term.^24^ For comparison, the normal Anderson–Evans structure is depicted as polyhedra in the inset to the left in a matching orientation. Color code: {WO_6_}, gray or purple, where the gray color indicates “nonclassical” fragments in “classical” structures; {TeO_6_}, pale yellow; C, gray; N, blue; O, red.

The so-called Mo or W storage protein (Mo/WSto), from *Azotobacter vinelandii*,^23^ which can store more than 100 metallic addenda atoms, can be viewed as a “reactor” for protein-supported POM cluster synthesis. “Nonclassical” POM structures, which are formed spontaneously and temporarily during protein crystallization, are selectively stabilized by the protein matrix. The monomeric orthomolybdate [Mo^VI^O_4_]^2–^ or orthotungstate [W^VI^O_4_]^2–^ form POM archetypes in protein pockets that have not been detected and synthesized under common inorganic conditions.^23^ The structures of these “nonclassical” POMs differ significantly from the “classical” structures of the same nuclearity and chemistry (Figure 2A), or their structural equivalents do exist but only as part of larger clusters (Figure 2B). As an example of a completely new archetype, [Mo_8_O_26_O(Glu)N(His)H_*n*_]^*n*−^ ({Mo_8_}; Figure 2A), is formed in MoSto [50 mM 3-(*N*-morpholino)propanesulfonic acid (MOPS)–NaOH, pH 6.5] and stabilized by binding to Hisα156–N_ε2_ and Gluα129–O_ε1_ fragments.^23c^ In the absence of protein, the formation of “classical” β-[Mo^VI^_8_O_26_]^4–^ (Figure 2A) occurs under more acidic conditions at pH < 5, indicating that the “rules” of pH-dependent condensation do not work in this bioreactor or that the protein microenvironment creates a “local pH” in the protein pocket. Examples of “nonclassical” POTs, which exist as subunits (“virtual building blocks”) of larger classical structures, have been detected in the protein pocket of WSto [1 M (NH_4_)_2_HPO_4_ and 0.1 M sodium citrate, pH 5.6].^23d^ The POT containing three W atoms with the assigned formula [W^VI^_3_O_10_H_*x*_N_3_]^(6–*x*)–^ (N atoms from the imidazole N_ε2_ atom of Hisα139) is the smallest one described so far and can be seen as a (formal) constituent of the well-known Keggin-type metatungstate [H_2_W^VI^_12_O_40_]^6–^^22^ (Figure 2B). The construction of small, atypical POM clusters could be used in the future to create new, larger architectures by using the Mo/WSto protein as a suitable “nano test tube”.^23f^ On the one hand, variation of the aqueous solutions could lead to different encapsulated POMs; on the other hand, modification of the relevant protein pockets can create new functions or patterns.

The protein environment can also transform preformed POM clusters into new structures during protein-supported POM crystal growth. The most prominent example here is modification of the Te-centered Anderson–Evans anion [Te^VI^W^VI^_6_O_24_]^6–^ (TEW),^24^ which is one of the most compact and rigid POM archetypes. An unprecedented organic hybrid [Te^VI^W^VI^_6_O_24_O_2_(Glu)]^7–^ (GluTEW)^25^ was formed via {W_6_} ring opening and the formation of W-μ_3_–O–C bonds (Figure 2C). This structure demonstrates the potential reactivity of the unprotonated μ_3_-O atoms (bridging O atom connected to two W and Te centers) of A-type Anderson POTs to undergo functionalization with alkoxy ligands. In a classical condensation reaction, it is not possible to graft alkoxy ligands onto the Anderson anions, which have unprotonated μ_3_-O atoms in their structures, as confirmed by single-crystal X-ray structure analysis.^24^ The unprecedented formation of the GluTEW hybrid under crystallization conditions indicates additional pathways for POM functionalization, which significantly alter the POM’s characteristics but may be less obvious from a pure inorganic point of view. Grafting of organic functionalities onto inorganic entities can increase the stability of POMs in biological media because of a decrease in the POM charge density.^26^ Moreover, a properly selected biologically active organic ligand can significantly improve the interaction of such POM hybrids with biomolecules.^8,27^

## 3 Problems Arising When Scientists Work in Intertwined Disciplines and Concepts for Overcoming Them

Scientists working at the intersection of disciplines have difficulties to overcome, which can usually be formulated as four questions:

(1) *What does interdisciplinary research include*? The simple blending of two disciplines without a multifaceted and integrated approach will not “solve the problems whose solutions are beyond the scope of a single discipline”.^2^ Nowadays we observe the tendency to present research as interdisciplinary, even if it is only slightly linked to another discipline. Truly interdisciplinary work involves the ability not only to combine knowledge from different disciplines but also to understand the fundamentals of the disciplines, to find out the relationship between their elements, and to decide what to do with critical unknowns.^28^

(2) *Academic restrictions on interdisciplinary training*. Conveying knowledge at the same high level in more than one discipline is a real challenge. Historically departments are organized around a single disciplinary core, which makes interdisciplinary education more complex.^29^ The rigid system of university organization, which is based on individual disciplines, often makes it difficult to support scientists with strong interdisciplinary research character.^30^

(3) *Each discipline speaks its own language*. Different disciplines represent different areas of knowledge and require expertise in using the “local” language, which makes it easier for scientists in one area to speak and learn with one another but also creates barriers against sharing knowledge with colleagues outside of the field. These “obstacles” often result from inadequate communication efforts on the part of one (or both) sending or receiving party (parties).

(4) *Difficulties in obtaining funding and ensuring high-quality peer review for interdisciplinary research results*. The number of scientists trained in more than one scientific field is too low, which negatively affects the implementation and revision of interdisciplinary work and projects. The perspective of any single discipline often does not suffice to comprehend, let alone rate, the (possible) outcome of truly interdisciplinary research and is therefore prone to underestimating both the challenges and benefits of such work. The great influence of interdisciplinary work on the future of science should encourage researchers to open their minds and think beyond one discipline.

**Scheme 1:**
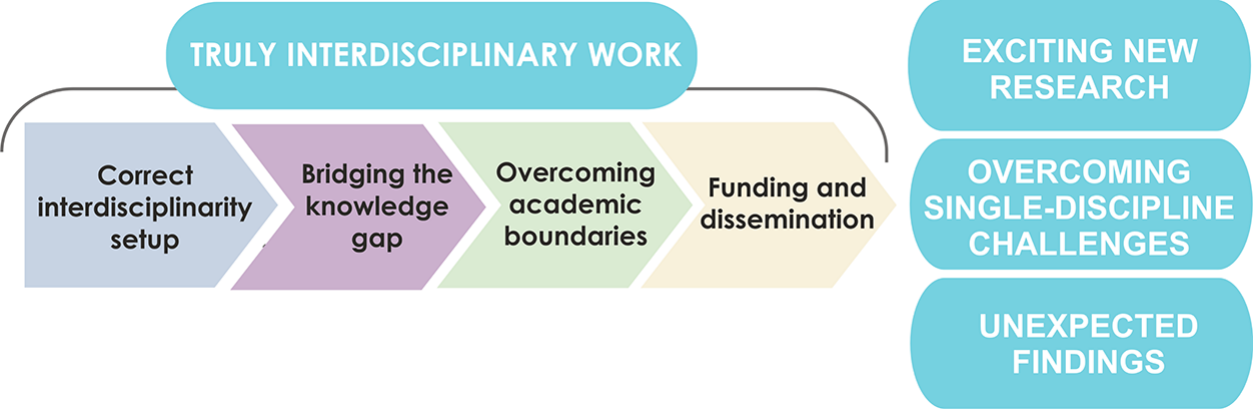
Essential Components and the Outcome of Truly Interdisciplinary Work

Key concepts for overcoming the challenges of interdisciplinary work are as follows (Scheme 1):

(1) *Support interdisciplinary research*. A multicomponent approach that takes the following points into account helps to implement interdisciplinary research tasks.
(2) *Overcoming academic boundaries*. It is obvious that dividing the time between two or more disciplines during academic studies is difficult to handle. Therefore, after gaining in-depth knowledge in one discipline, scientists should be encouraged to acquire knowledge in another discipline in order to be able to establish suitable interdisciplinary cross-connections. It is important to acquire new knowledge at each stage of an academic career, and the term “life-long-learning/training” is an important characteristic of interdisciplinary work. A change of the research area can be a disadvantage when applying for third-party funding because the mandatory preliminary results may be missing. Therefore, funding for researchers who want to grow into a new scientific field must be available to broaden the scientific horizon at all stages of an academic career. A scientist who is working at the intersection of disciplines should not be perceived as a person sitting between chairs but as a valuable researcher who has outgrown an established area. Universities should promote the integration of disciplinary perspectives to tackle complex problems even more, and the filling of scientific positions in interdisciplinary areas is necessary to develop new research fields.
(3) *Overcoming language barriers*. In order to be able to work on interdisciplinary tasks with a research partner, each scientist has to become proficient in the language of the other discipline to a certain extent. Joint project planning, taking into account the particularities of the individual disciplines, instead of two separate, parallel projects that are combined after completion, is the key to new discoveries and an understanding of complex phenomena.
(4) *Funding and dissemination of interdisciplinary results*. The growing number of journals covering interdisciplinary research is a useful tool. It is desirable that interdisciplinary research should be reviewed by scientists proficient in interdisciplinary research, the number of whom will hopefully increase in the future.^31^

This perspective is written with the expectation of encouraging chemists and biologists with diverse backgrounds to expand their areas of interest and share their knowledge across disciplines, thereby fostering the successful development of truly interdisciplinary work and advancing inorganic biochemistry.
